# High genetic diversity and low population differentiation of a medical plant *Ficus hirta* Vahl., uncovered by microsatellite loci: implications for conservation and breeding

**DOI:** 10.1186/s12870-022-03734-2

**Published:** 2022-07-12

**Authors:** Yi Lu, Jianling Chen, Bing Chen, Qianqian Liu, Hanlin Zhang, Liyuan Yang, Zhi Chao, Enwei Tian

**Affiliations:** 1 School of Traditional Chinese Medicine, Southern Medical University, Guangzhou, 510515 China; 2 Affiliated Cancer Hospital & Institute of Guangzhou Medical University, Guangzhou, 510095 China; 3 Department of Landscape Plants and Ornamental Horticulture, College of Landscape Architecture, Zhejiang Agriculture & Forestry University, Hangzhou, 311300 People’s Republic of China; 4 Zhejiang Provincial Key Laboratory of Germplasm Innovation and Utilization for Garden Plants, Zhejiang Agriculture & Forestry University, Hangzhou, 311300 People’s Republic of China; 5 Guangdong Provincial Key Laboratory of Chinese Medicine Pharmaceutics, Guangzhou, 510515 China; 6 Guangdong Provincial Engineering Laboratory of Chinese Medicine Preparation Technology, Guangzhou, 510515 China

**Keywords:** *Ficushirta*, Simple sequence repeat (SSR), Genetic diversity, Genetic structure, Breeding, Conservation

## Abstract

**Background:**

Wuzhimaotao (Radix Fici Hirtae) originates from the dry root of *Ficus* *hirta* (Moraceae), which is widely known as a medical and edible plant distributed in South China. As the increasing demand for Wuzhimaotao, the wild *F.* *hirta* has been extremely reduced during the past years. It is urgent to protect and rationally develop the wild resources of *F.* *hirta* for its sustainable utilization. However, a lack of genetic background of *F.* *hirta* makes it difficult to plan conservation and breeding strategies for this medical plant. In the present study, a total of 414 accessions of *F.* *hirta* from 7 provinces in southern China were evaluated for the population genetics using 9 polymorphic SSR markers.

**Results:**

A mean of 17.1 alleles per locus was observed. The expected heterozygosity (*H*e) varied from 0.142 to 0.861 (mean = 0.706) in nine SSR loci. High genetic diversity (*H*_e_ = 0.706, ranged from 0.613 to 0.755) and low genetic differentiation among populations (*G’*_ST_ = 0.147) were revealed at population level. In addition, analysis of molecular variance (AMOVA) indicated that the principal molecular variance existed within populations (96.2%) was significantly higher than that among populations (3.8%). Meanwhile, the three kinds of clustering methods analysis (STRUCTURE, PCoA and UPGMA) suggested that the sampled populations were clustered into two main genetic groups (K = 2). Mantel test showed a significant correlation between geographic and genetic distance among populations (*R*^2^ = 0.281, *P* < 0.001). Pollen flow, seed flow and/or geographical barriers might be the main factors that formed the current genetic patterns of *F.* *hirta* populations.

**Conclusions:**

This is a comprehensive study of genetic diversity and population structure of *F.* *hirta* in southern China. We revealed the high genetic diversity and low population differentiation in this medicinal plant and clarified the causes of its current genetic patterns. Our study will provide novel insights into the exploitation and conservation strategies for *F.* *hirta*.

## Background

*Ficus* *hirta* Vahl. is a perennial deciduous shrub or small tree which widely distributed in Southeast Asia, Northeast India and South China [1]. In south China, especially for Guangdong, Guangxi and Hainan provinces, the dry root (Radix Fici Hirtae) of this species has been used as an ethnic herbal medicine with the effects of dispelling the dampness and invigorating the spleen, nourishing the lung for a long time in Yao and Zhuang National Regions [2–4]. It contains many active compounds such as coumarins, volatile oils, amino acids, saccharides, steroids, phenolics, flavonoids and phenylpropanoids along with multiple therapeutic effects such as antitumor, antifungal and hepatoprotective which were confirmed by modern pharmacological researches [5, 6]. In addition to the medicinal values, it is also an edible healthy food with special aroma that has been widely used in food industry. Therefore, Radix Fici Hirtae in both Pharmaceutical and food industry is in great demand every year. It is worth noting that Good Agricultural Practice (GAP) bases [7] for Radix Fici Hirtae have already been built in several cities of Guangdong, Guangxi and Hainan provinces, but the chief commodity circulated in the market still sources from the wild *F.* *hirta*. The wild resources of this species are continuously shrinking due to over-exploitation and environmental destruction mediated by human beings [8]. It is urgent to protect and rationally develop the wild resources of *F.* *hirta* for its sustainable utilization.

Researches on genetic diversity and population structure of medicinal plants are the premise and fundamental issues for the protection and utilization of medicinal plant germplasm resources. The protection of medicinal plant germplasm resources is to protect its genetic diversity and evolutionary potentials [9, 10]. Although *F.* *hirta* have been successfully cultivated in some areas, to some extent, which also relieved the pressure on nature resources, long term cultivation and directional selection of this species may also cause the loss of genetic diversity, further lose their ability of resisting the epidemic of pests and diseases [11, 12]. Therefore, it is very important to protect the genetic diversity of this species. Up to date, however, we have known very little about the genetic background of wild *F.* *hirta* resources in China, which makes it difficult to develop breeding and conservation strategies for this species. Thus, it is of great significance to study the genetic diversity and population structure of wild *F.* *hirta* throughout all distribution areas in China.

In recent years, simple sequences repeat (SSR) marker has become one of the most popular tools for analyzing genetic diversity and population structure, gene flow, phylogenetics in conservation genetics, because of its co-dominant inheritance, high polymorphism, high abundance, selective neutrality and excellent repeatability [13]. During the pre-experiment stage, we had screened the highly polymorphic SSRs in previous studies [14–17]. We obtained nine polymorphic SSR loci and conducted the population genetics of *F.* *hirta*. In this study, we aimed: (1) to systematically reveal genetic diversity and population structure of *F.* *hirta* from 18 populations located in 7 provinces in South China; (2) to clarify the causes of the current genetic patterns of this species; (3) to provide references for the conservation and breeding of germplasm resources of *F.* *hirta*.

## Results

### Polymorphism for the nine SSR loci

A total of 154 alleles were amplified across 414 individuals from 18 populations. The total number of alleles per locus (*N*_a_) varied greatly among loci, from 7 to 23 alleles (mean = 17.1), while the number of effective alleles per locus (*N*_e_) ranged from 1.23 to 7.9 (mean = 4.9). The observed heterozygosity (*H*_o_) ranged among loci from 0.179 to 0.905 (mean = 0.638), while the expected heterozygosity (*H*_e_) ranged from 0.142 to 0.861 (mean = 0.706) (Table 1). No evidence for stuttering, large allele dropout or null alleles was found using MICRO-CHECKER.

**Table 1:** Statistical polymorphism of 9 SSRs on 414 samples across 18 populations of *Ficus* *hirta* in China

Loci	*N*_a_	*N*_e_	*H*_o_	*H*_e_
FH3	11	2.945	0.621	0.642
FH7	16	6.818	0.905	0.843
FH10	14	3.038	0.591	0.641
FH14	7	1.227	0.179	0.142
FH21	21	4.256	0.755	0.759
FH23	21	7.899	0.819	0.861
FH47	21	6.827	0.533	0.845
FS4-11	20	5.075	0.712	0.792
MFC1	23	5.998	0.628	0.826
Total	154	/	/	/
Mean	17.111	4.898	0.638	0.706

*N*a: The total number of observed alleles per locus, *N*e: The effective number of alleles, *H*o: Observed heterozygosity, *H*e: Expected heterozygosity

### Population genetic diversity

At population level, genetic diversity indices (in terms of *N*_a_, *N*_e_, *H*_o_, *H*_e_, *F*_IS_) varied across populations of *F.* *hirta*. The *N*_a_ per population varied from 5.4 (ZJ) to 9.4 (SD), with a mean of 7.8, while the *N*_e_ ranged from 3.7 (ZJ) to 6.2 (SD) (mean = 4.9). The mean *H*_e_ and *H*_o_ across all populations ranged from 0.525 (WN) to 0.731 (ZP) and 0.613 (ZJ) to 0.751 (SD), with means of 0.638 and 0.706, respectively. The private allele richness (*P*_Ar_) of each population ranged from 0 (WN, ZP, SX, ND) to 0.778 (NN), with a mean of 0.228. All populations showed significant (*P* < 0.05) deviations from HWE that were not significant after sequential Bonferroni correction. And no bottleneck effects were detected under the TPM model (Table 2).

**Table 2:** Genetic diversity for each population of *Ficus* *hirta*

Pop	*N*_a_	*N*_e_	*H*_o_	*H*_e_	*F*_IS_	*P*_Ar_	TMP probability
ZJ	5.444	3.722	0.570	0.613	-0.054	0.333	0.434
LS	7.667	4.382	0.614	0.681	0.116	0.333	0.530
WN	6.556	3.768	0.525	0.650	0.345	0.000	0.564
SD	9.444	6.205	0.679	0.751	-0.063	0.222	0.476
HC	8.667	4.807	0.619	0.695	-0.068	0.222	0.566
HZ	8.444	5.140	0.641	0.729	-0.005	0.222	0.524
NN	8.667	5.185	0.574	0.707	-0.056	0.778	0.201
JY	8.889	5.828	0.631	0.735	-0.070	0.111	0.527
LD	8.444	5.117	0.673	0.712	-0.077	0.333	0.565
SG	9.111	5.974	0.647	0.722	0.030	0.333	0.427
SZ	8.222	5.765	0.709	0.755	0.018	0.444	0.469
HY	7.889	5.449	0.559	0.716	-0.004	0.111	0.210
ZP	7.111	4.341	0.731	0.691	-0.089	0.000	0.476
LP	8.111	4.924	0.709	0.715	-0.196	0.333	0.487
MZ	7.222	4.627	0.686	0.715	-0.048	0.111	0.068
GZ	6.889	4.407	0.658	0.699	-0.063	0.222	0.227
SX	6.667	3.967	0.621	0.687	-0.128	0.000	0.220
ND	7.222	4.559	0.639	0.728	-0.069	0.000	0.223
mean	7.815	4.898	0.638	0.706	-0.027	0.228	0.400

*N*_a_: The observed number of allele for each population over loci; *N*_e_: The effective number of alleles; *H*_o_: Observed heterozygosity; *H*_e_: Expected heterozygosity; *F*_IS_: Inbreeding coefficient; *P*_Ar_: Private allele richness; Departure from HWE: *P < 0.05 (significant); ** *P* < 0.001 (extremely significant), no population showed significant (*P* > 0.05) deviation from HWE after sequential Bonferroni correction

### Population structure and genetic differentiation

**Fig. 1:**
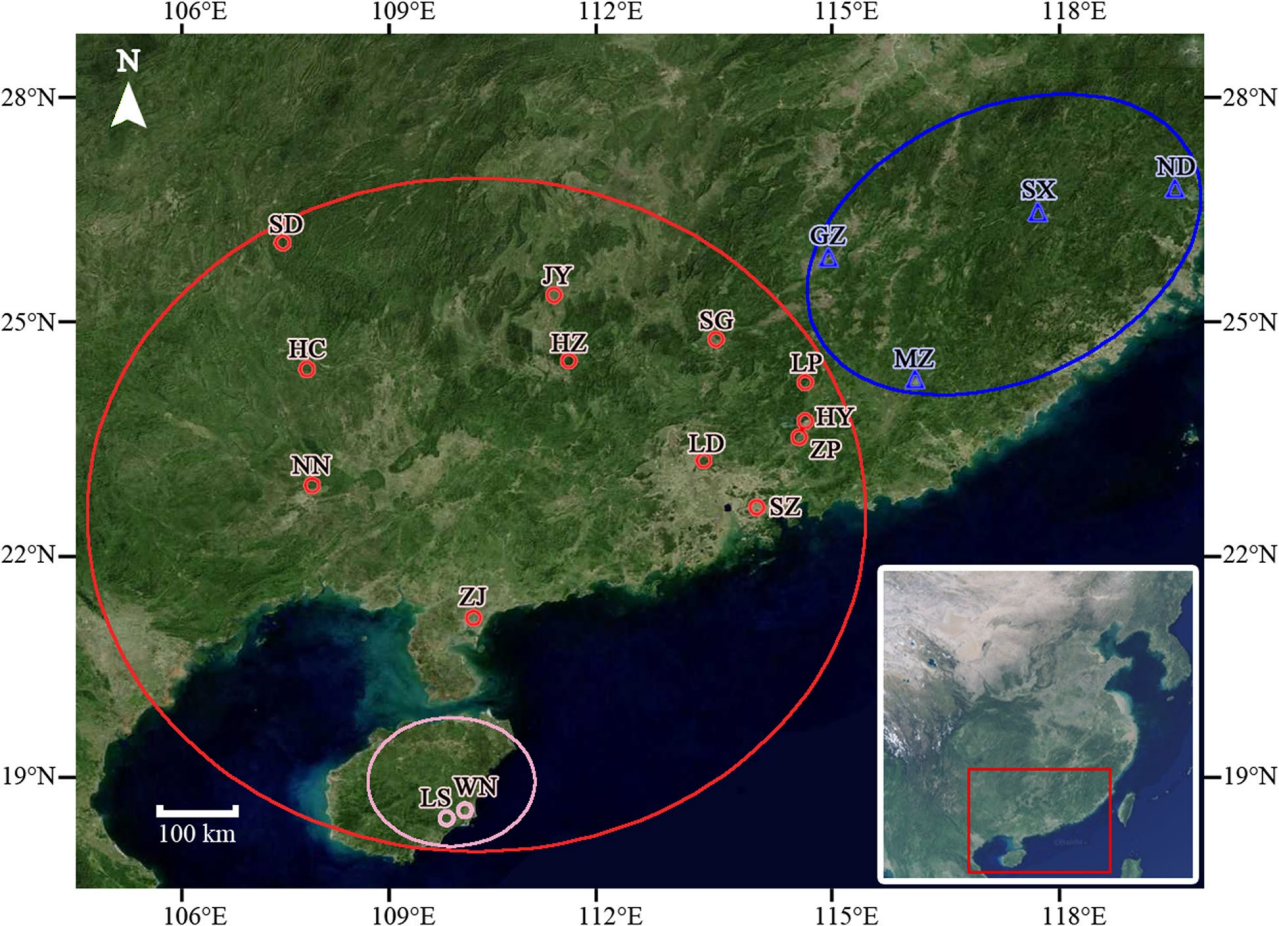
Sampling geographic distribution of *F.* *hirta* in China and its two main clusters (Mid-south Cluster and Eastern Cluster marked by red circles and blue triangles) according to the STRUCTURE results. The map was drawn by the authors with reference to Google Maps. The map can be found at https://maps.google.com/

**Fig. 2:**
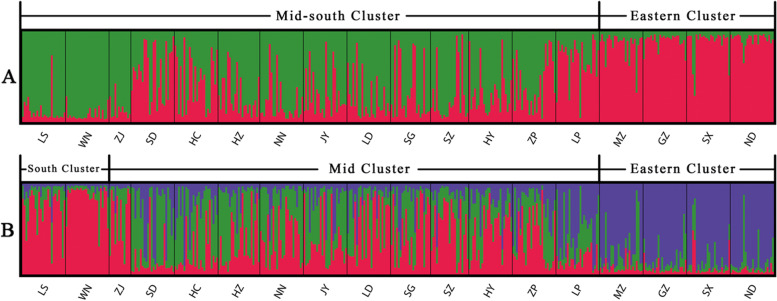
STRUCTURE analysis of *F.* *hirta* in China based on 9 nSSRs: **A** bar plots of the membership probabilities at K = 2; **B** the Mid-south cluster were subdivided into mid cluster and South cluster at K = 3

Based on maximum delta K (ΔK) values, the optimal number of genetic clusters equaled two (K = 2). The 18 populations formed in to two clusters. The cluster I included 4 populations from eastern region (MZ, GZ, ND and SX), while the cluster II included 14 populations in mid-south region (ZJ, WN, LS, SD, HC, HZ, NN, JY, LD, SG, SZ, HY, ZP and LP) (Figs. 1 and 2A). A tentative cluster approach of K = 3 was also demonstrated below where the mid-south cluster was further divided into two sub-clusters: the mid sub-cluster (SD, HC, HZ, NN, JY, LD, SG, SZ, HY, ZP and LP) and southern sub-cluster (ZJ, LS and WN) (Figs. 1 and 2B).

**Fig. 3:**
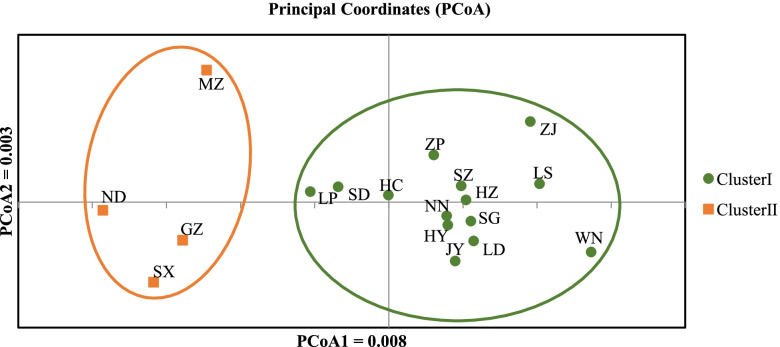
Principal coordinate analysis (PCoA) of 18 *F.* *hirta* populations. Two axes from the PCoA explain 51.21% of the total observed variation

**Fig. 4:**
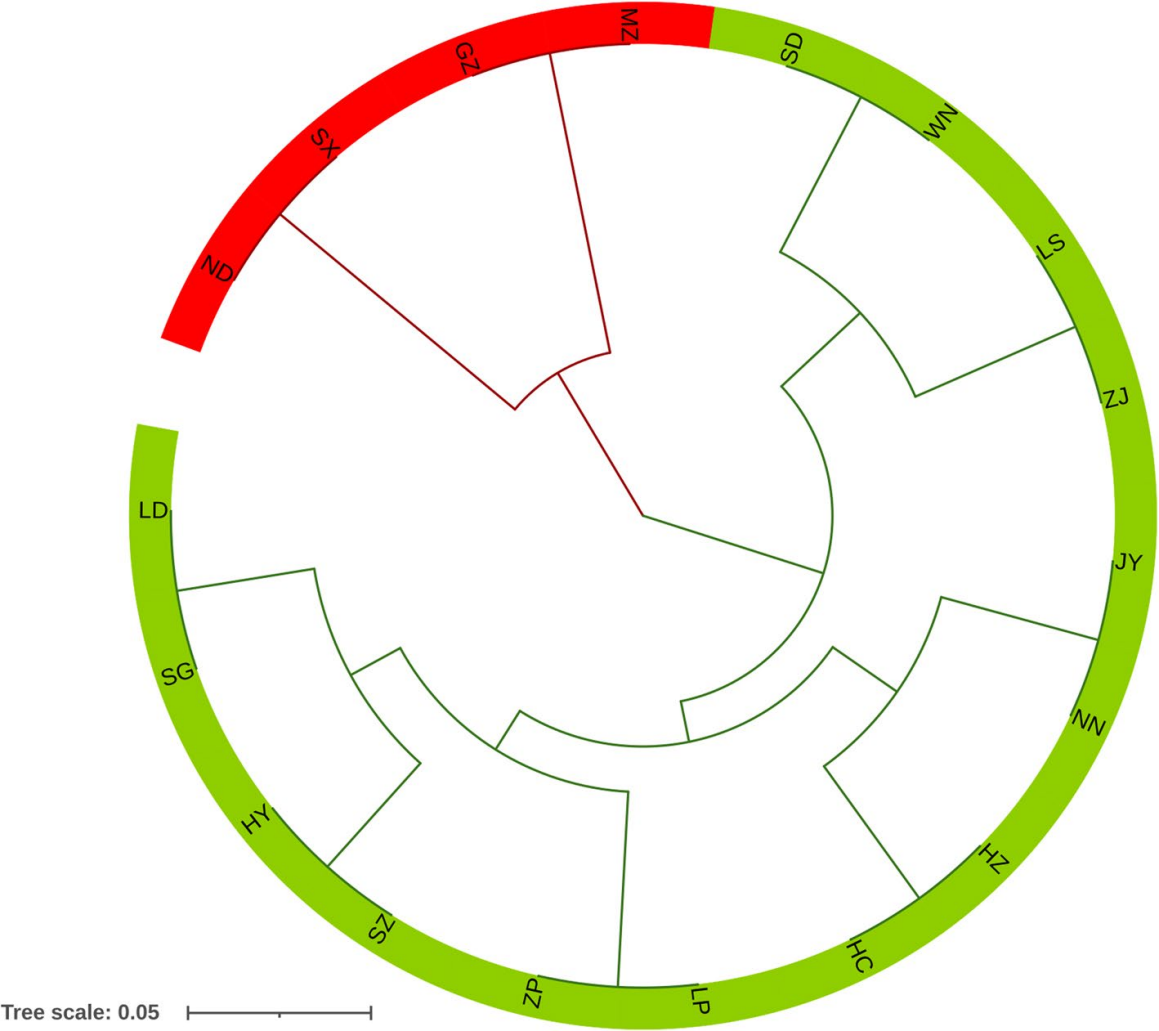
Unweighted pair group method with an arithmetic mean (UPGMA) analysis of 18 *F.* *hirta* populations

The PCoA results were consistent with the STRUCTURE analysis. The cumulative percentage variance attributable to the first three principal coordinate axes was 60.2% (axis 1–36.6%, axis 2–14.6% and axis 3–9.0%) (Fig. 3). The UPGMA tree showed two main branches, which was in agreement with the PCoA and STRUCTURE analysis (Fig. 4).

**Fig. 5:**
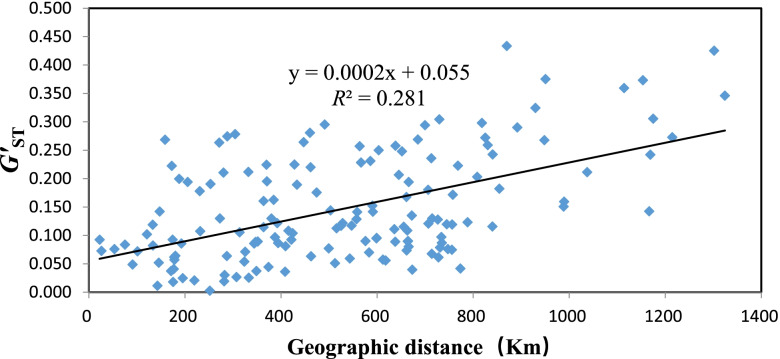
Isolation by distance with Mantel test for *F.* *hirta*. The regression of the standardized genetic differentiation *G’*_ST_ vs the geographic distance (Km) was highly significant for the SSR data (*P* < 0.001, *R*^2^ = 0.281)

Pairwise estimates of *G*’_ST_-values ranged from -0.004 (between SG and HY) to 0.434 (between WN and MZ) with a mean of 0.147 (Table 3). Across all the populations, AMOVA demonstrated that most genetic variation occurred within populations of *F.* *hirta* (96.2%), while the variation among populations was only 3.8% (Table 4). Within the cluster, all geographic clusters had higher genetic variations within populations than that among populations. The genetic differentiation among clusters was 3.0% (two clusters) and 3.5% (three clusters). The genetic variation among the sampled populations from the mid-south cluster was 2.9%, in which the value of mid and south cluster was 2.0% and 1.1%, respectively. While the genetic variation among populations from the eastern cluster was 2.4%. Mantel test presented a significant pattern that genetic distance increase with the geographical distance between populations of *F.* *hirta* (*P* < 0.001, *R*^2^ = 0.281) (Fig. 5).

**Table 3:** Pairwise *G*’_ST_ value among 18 populations of *Ficus* *hirta*

	**ZJ**	**LS**	**WN**	**SD**	**HC**	**HZ**	**NN**	**JY**	**LD**	**SG**	**SZ**	**HY**	**ZP**	**LP**	**MZ**	**GZ**	**SX**	**ND**
ZJ	0.000																	
LS	0.019	0.000																
WN	0.130	0.073	0.000															
SD	0.142	0.116	0.242	0.000														
HC	0.104	0.090	0.194	0.041	0.000													
HZ	0.087	0.040	0.168	0.081	0.037	0.000												
NN	0.064	0.077	0.144	0.089	0.038	0.097	0.000											
JY	0.176	0.075	0.120	0.115	0.086	0.049	0.093	0.000										
LD	0.163	0.056	0.095	0.111	0.117	0.108	0.117	0.027	0.000									
SG	0.113	0.042	0.119	0.070	0.059	0.025	0.090	0.021	0.018	0.000								
SZ	0.108	0.057	0.153	0.087	0.089	0.026	0.096	0.036	0.072	0.003	0.000							
HY	0.122	0.076	0.128	0.079	0.080	0.054	0.108	0.044	0.052	-0.004	0.011	0.000						
ZP	0.051	0.062	0.180	0.097	0.090	0.071	0.115	0.130	0.119	0.062	0.102	0.092	0.000					
LP	0.141	0.123	0.223	0.068	0.073	0.106	0.135	0.089	0.092	0.082	0.086	0.076	0.084	0.000				
MZ	0.269	0.290	0.434	0.182	0.203	0.281	0.298	0.295	0.279	0.264	0.211	0.269	0.222	0.142	0.000			
GZ	0.294	0.268	0.325	0.130	0.121	0.195	0.172	0.161	0.212	0.200	0.224	0.178	0.191	0.057	0.194	0.000		
SX	0.375	0.306	0.373	0.151	0.159	0.248	0.212	0.207	0.228	0.220	0.257	0.225	0.264	0.122	0.275	0.030	0.000	
ND	0.359	0.346	0.425	0.143	0.242	0.259	0.273	0.272	0.305	0.258	0.236	0.231	0.250	0.129	0.189	0.064	0.064	0.000

**Table 4:** Analyses of molecular variance (AMOVAs) for 18 *Ficus* *hirta* populations assessed with 9 microsatellite loci

	Source of Variation	d.f	SS	Variance components	Percentage of variation (%)	*φ*_ST_	*P*
Species	Among populations	17	37.880	0.032	3.8	0.038	*P* < 0.001
	Within populations	808	650.072	0.804	96.2		
The mid-south cluster (ZJ, WN, LS, SD, HC, HZ, NN, JY, LD, SG, SZ, HY, ZP, LP)	Among populations	13	12.471	0.012	2.9	0.029	*P* < 0.001
	Within populations	620	251.144	0.405	97.1		
The eastern cluster (MZ, GZ, SX, ND)	Among populations	3	5.365	0.020	2.4	0.024	*P* < 0.001
	Within populations	188	152.917	0.813	97.6		
Two clusters	Among populations	1	8.442	0.026	3.0	0.030	*P* < 0.001
	Within populations	824	679.510	0.825	97.0		
The mid cluster (ZJ, SD, HC, HZ, NN, JY, LD, SG, SZ, HY, ZP, LP)	Among populations	11	8.715	0.009	2.0	0.020	*P* < 0.001
	Within populations	526	216.415	0.411	98.0		
The south cluster (WN, LS)	Among populations	1	1.687	0.016	1.10	0.011	*P* < 0.005
	Within populations	94	102.104	1.086	98.9		
Between Hainan island and mainland China	Among populations	1	7.698	0.041	4.7	0.047	*P* < 0.001
	Within populations	824	680.253	0.826	95.3		
Three clusters	Among populations	2	14.233	0.030	3.5	0.035	*P* < 0.001
	Within populations	825	675.393	0.819	96.5

## Discussion

### High Genetic diversity and low population differentiation of Ficus hirta

The roots of *F.* *hirta* have been used as important herb medicine to prevent and treat many human diseases, such as asthma, stomachache, rheumatism and irregular menstruation in China for hundreds of years [18]. Given the dramatically increasing demand for the root materials, *F.* *hirta* are under over-exploitation, and furthermore, the ecological environments of the original distribution area are under destruction year by year [8]. In order to make sustainable use of this medical resources, effective management measures for the conservation are necessary to be taken to further protect the wild *F.* *hirta* plants. Generally, genetic diversity underlies adaptation and evolution of plants, which allows for mitigating against various stresses of the changing environments [19, 20]. Therefore, it is necessary to study the genetic variation of *F.* *hirta* for proposing the conservation strategies and breeding programs.

In this study, all of the nine loci used are highly informative with an average of 17.1 alleles per locus, which is higher than previous report which was made by Zheng et al. [17], with an average of 5.6 alleles per locus. The differences may refer to the number of loci and samples sizes used [21]. A variety of genetic diversity indices revealed that the *F.* *hirta* in China maintained abundant genetic variation at species level (*H*_e_ = 0.706, *H*_o_ = 0.638). Compared with previous studies on congeneric dioecious fig species, the genetic diversity of *F.* *hirta* was higher than those (*H*_e_ = 0.370—0.663) of *F.* *carica*, *F.* *hispida*, *F.* *heterostyla*, *F.* *squamosa* and *F.* *pumila* [22, 23]*.* At population level, all populations also maintained high genetic diversity (*H*_e_ > 0.6). For the high genetic variation of *F.* *hirta*, according to our observation, the pollinator (*Blastophaga* *javana*) of this species can pollinate almost all a year round (except for severe cold winter). The long pollinating period combined with the monsoon climate in southern China may be conducive to the wide-ranging dissemination of pollen and seed [24, 25]. On the other hand, the selection for those highly polymorphic loci may lead to an ascertainment bias of higher heterozygosity [26]. Partially, it may also be related to the wide distribution area and different surrounding environment (coastal or landlocked habitats). Adaptive evolution may result in a rich gene pool and increase genetic variation [19, 27]. Many examples, such as *Phyllanthus* *emblica*, *Trifolium* *repens*, which have proved outbreeding species, were trend to have higher genetic diversity [19, 28, 29]. Indeed, our analysis of the inbreeding coefficient (average *F*_IS_ = -0.027) of *F.* *hirta* populations suggested that most of populations, except for the Hainan island populations (LS, WN), were consistent with the low inbreeding level of congeneric species (average *F*_IS_ = -0.056—0.054) [30]. The *F*_IS_ was relatively high in WN population (0.345) and LS population (0.116), indicating heterozygotes deficiency in both populations, which could be explained by the large number of plant individuals of *F.* *hirta* occupied within Hainan island. As high plant density of *F.* *hirta* will shorten the pollinating distance of the pollinators between figs. Another plausible explanation for the lower genetic diversity with island populations is the special geographical distance isolated by Qiongzhou strait (The Hainan island has a geographical span of 30 km with China mainland), which hinders the gene exchanges between islands and Chinese mainland. High inbreeding occurring within Hainan island populations may also explain the relative low genetic diversity of WN and LS populations to other populations in China mainland.

STRUCTURE, PCoA and UPGMA clustering analysis all assigned the 18 populations into two clusters, of which included the populations of ZJ, LS, WN, SD, HC, HZ, NN, JY, LD, SG, SZ, HY, ZP, LP in cluster I and MZ, GZ, SX, ND in cluster II. The two clusters distributed at the mid-south region and eastern region of South China respectively, and the genetic structure was largely related with geographical isolation of *F.* *hirta*. The correlation between the geographical distance and genetic divergence of *F.* *hirta* populations were further supported by an IBD analysis with Mantel test (*R*^2^ = 0.281, *P* < 0.001). This may be related to the geographical isolation barrier of Luoxiao Mountains and Nanling Mountains between the mid-south and northeast populations, diminishing the gene flow between the above two clusters (which can also be found in other species like *Gynostemma* *pentaphyllum* [21], *Eomecon* *chionantha* [31] and *Cercis* *chuniana* [32]. Although there was a significant IBD patterns of *F.* *hirta*, we found a relatively low genetic differentiation in *F.* *hirta* (*G’*_ST_ = 0.147) (Table 3). Molecular analysis of variance (AMOVA) also showed that the variation of *F.* *hirta* coming from the inter-population variation is only 3.8% (Table 4). Generally, gene flow resulted from migration of pollen and seeds, plays a major role in preventing genetic differentiation among populations and fostering the conservation of genetic diversity [33]. Previous study has suggested that the pollinator of *F.* *hirta* can pollinate over long distance, which results in a long-distance genetic migration and creates a wide gene pool [30]. In addition, fruit-eating animals like birds, monkeys and bats etc. also play a role in fig seed dispersal [34, 35]. Meanwhile, according to our observations, the large effective population size and relatively continuous distribution of *F.* *hirta* may further prevent the loss of novel alleles, and consequently maintain the gene flow and genetic diversity. However, the genetic variation of *F.* *hirta* between Hainan island and mainland China was up to 4.7% (Table 4). Although it is suggested that the pollinator of fig can pollinate through a long-distance [36, 37], the higher genetic differentiation found in Hainan province might be partially result from the geographic barrier of Qiongzhou straight. As evidenced from *F.* *pumila* [38] and *Oryza* *ruffipogon* [39] that geographic isolation may decrease the gene flow, which could counteract with natural selection, as a result, causing stronger genetic drift and the populations would generate genetically differentiated gradually.

In this part, we confirmed that the *F.* *hirta* from South China we investigated had the genetic patterns of low differentiation among populations and high diversity within populations.

### Conservation and breeding strategy for Ficus hirta

Although *F.* *hirta* is widely distributed in the Subtropical and tropical China, a rising demands on the roots of *F.* *hirta* for medicine and food industry has resulted in a severe decline of this plant resources. GAP bases of this traditional herbal medicine have been established in Guangdong, Guangxi and Hainan provinces in recent years, but it’s still unable to meet the needs of the medicine and food market. Nevertheless, the progress in breeding and conservation of *F.* *hirta* has been slow because of lacking the genetic background knowledge. Our large-scale population genetics study of *F.* *hirta* showed the highest genetic diversity found in SD and SZ populations, while ZJ population was found to be the lowest within the 18 populations. The private alleles were found in most populations (14/18), implicating the specific genes or haplotypes in these populations. The highest *P*_Ar_ was found in NN (0.778), followed by SZ (0.444), while no private allele was detected in WN, SX, ND and a cultivated population ZP. That indicated these populations might maintain a subset of the total genetic diversity present in their wild ancestors.

According to genetic structure of *F.* *hirta*, it is necessary for us to preserve the two detected clusters and also take into account the populations with high levels of genetic diversity along with those with private alleles (except WN, ZP, SX, ND). In addition to in situ conservation, ex situ germplasm collection efforts should focus on as many populations as possible for breeding and resources protection. Particularly, individuals from SD, SZ populations in Mid-South cluster, which have higher level of genetic diversity than other populations, and those from NN population which are rich in private alleles also should be taken into consideration (Given the low *φ*_ST_ values, because individuals in these populations may represent a major components of genetic diversity of *F.* *hirta*) [40]. Finally, our investigation on the genetic background of *F.* *hirta* is expected to be further applied in commercial planting (breeding practice), so as to improve its yield and quality and ensure the sustainable utilization of the wild plant resources.

## Conclusions

In this study, we used nine highly polymorphic SSR markers to study the genetic diversity and population structure of *F.* *hirta* in southern China. Our results showed: (a) *F.* *hirta* maintained relatively high genetic variation and most of the populations contain a number of private alleles and although there was a relatively low genetic differentiation among populations of *F.* *hirta*, it still performed a significant IBD pattern. (b) The population genetic clustering suggested that the 18 populations formed into two distinctive clusters which just corresponded to their geographical distribution (mid-south cluster and eastern cluster), of which the mid-south cluster could further be subdivided into mid cluster and south cluster. It is inferred that the formation of current genetic patterns in *F.* *hirta* was mainly resulted from pollen flow, seed flow and geographical barriers. (c) Although our results showed that the genetic resources of *F.* *hirta* in China were relatively abundant, we should still concern about the large consumption of its wild resources and take actions to protect this eatable and medicinal plant. Our results will provide molecular biological basis for variety improvement, germplasm conservation and establishment of Core Germplasm Bank for *F.* *hirta*.

## Methods

### Plant materials

We collected the specimens of *F.* *hirta* from 18 populations involving 7 provinces which almost ranged all its distribution areas in South China (Fig. 1, Table 5). Most of samples were in wild growth, except 24 individuals sourced from ZP population, which were cultivated in Heyuan city of Guangdong province. Samples collection protocols are as follow: in each population, the distance between each collected individual plant was over 20 m, which aimed to avoid multiple samples from the same clone. Totally, we obtained 414 samples (average 23 individuals for each population with a range of 12 to 24) and fresh tender leaves of each individual were dried in silica gel for DNA extraction. All voucher specimens were morphologically identified by associate professor Enwei Tian from School of Traditional Chinese Medicine, Southern Medical University (SMU) and deposited at the herbarium of SMU. The above specimens we collected were not collected in nature reserves and this species has also not been listed in national key protected plants. We collected the samples without any required permissions. Our field investigations and experimental studies complied with local legislation, national and international guidelines. The authors also complied with the Convention on the Trade in Endangered Species of Wild Fauna and Flora.

**Table 5:** Locality and voucher specimens information for each population of *Ficus* *hirta* in South China

Population ID	Locality	Specimen Voucher no	Geographical coordinates	Sample sizes
ZJ	Zhanjiang, Guangdong	20,201,003-ZJ (ZJ1 ~ ZJ12)	N: 21.168°; E: 110.338°	12
LS	Lingshui, Hainan	2,020,718-LS (LS1 ~ LS24)	N: 18.663°; E: 109.931°	24
WN	Wanning, Hainan	2,020,717-WN (WN1 ~ WN24)	N: 18.719°; E: 110.172°	24
SD	Sandu, Guizhou	2,020,721-SD (SD1 ~ SD24)	N: 25.968°; E: 107.846°	24
HC	Hechi, Guangxi	2,020,720-HC (HC1 ~ HC24)	N: 24.402°; E: 108.142°	24
HZ	Hezhou, Guangxi	2,020,723-HZ (HZ1 ~ HZ23)	N: 24.519°; E: 111.587°	23
NN	Nanning, Guangxi	2,020,719-NN (NN1 ~ NN24)	N: 22.866°; E: 108.237°	24
JY	Jiangyong, Hunan	2,020,724-JY (JY1 ~ JY24)	N: 25.321°; E: 111.402°	24
LD	Guangzhou, Guangdong	20,201,001-LD (LD1 ~ LD24)	N: 23.206°; E: 113.357°	24
SG	Shaoguan, Guangdong	20,201,004-SG (SG1 ~ SG24)	N: 24.778°; E: 113.499°	22
SZ	Shenzhen, Guangdong	20,201,007-SZ (SZ1 ~ SZ21)	N: 22.575°; E: 114.07°	21
HY	Heyuan, Guangdong	20,201,005-HY (HY1 ~ HY24)	N: 23.738°; E: 114.662°	24
ZP	Heyuan, Guangdong	20,201,006-ZP (ZP1 ~ ZP24)	N: 23.543°; E: 114.608°	24
LP	Lianping, Guangdong	20,201,005-LP (LP1 ~ ZP24)	N: 24.219°; E: 114.667°	24
MZ	Meizhou, Guangdong	20,201,006-MZ (MZ1 ~ MZ24)	N: 24.251°; E: 116.121°	24
GZ	Ganzhou, Jiangxi	2,020,727-GZ (GZ1 ~ GZ24)	N: 25.794°; E: 114.991°	24
SX	Shaxian, Fujian	2,020,728-SX (SX1 ~ SX24)	N: 26.387°; E: 117.744°	24
ND	Ningde, Fujian	2,020,729-ND (ND1 ~ ND24)	N: 26.637°; E: 119.53°	24
Total				414

### DNA extraction and PCR amplification

Total genomic DNA was isolated using a modified cetyltrimethyl ammonium bromide (CTAB) method [41]. The integrity and quality of the DNA were first checked on 1.5% agarose gel and then quantified using Nanodrop (ND2000C, Thermo Fisher Scientific). A working concentration of 20 ng/μl DNA stock was prepared for all the 414 samples of *F.* *hirta* and stored at -20 °C for further study.

Nine highly polymorphic SSR primers were screened in previous studies [14–17] and were chosen for PCR amplification. All forward primers were 5' end-labeled with fluorescent dyes (FAM, HEX or TAMRA) (Table 6). PCR amplification reactions were performed in a final volume of 20 μl containing 2 μl template DNA (20 ng/μl), 3 μl of 10 × PCR buffer (contains Mg^2+^), 2 μl of 10 mM dNTPs, 1 μl of each primer (10 μM), 1 U of Taq DNA polymerase (Takara., Kyoto, Japan) and 10.8 μl ddH_2_O water. The reaction was run using a Thermal Cycler (A300, LongGene) with the following program: 94℃for 5 min, 35 cycles of 45 s denaturation at 94℃, then 60 s at a primer-specific annealing temperature at the range of 50℃ ~ 60℃, and 45 s extension at 72℃, followed by 7 min extension at 72℃, and then a final holding temperature at 4℃. PCR amplification products were separated on 1.5% agarose gels, and visualized under UV light and further sent to Thermo Fisher Technology Co., Ltd (Guangzhou, China) for sequencing. Liz 500 (Applied Biosystems) was used as internal standard, and Peakscanner v1.0 was used to read SSRs genotypes data.

**Table 6:** Information of nine pairs of selected polymorphic SSR primers

SSR primers (Fluorescent label)	Primer sequences (5′–3′)	Repeat units	Length (bp)	Literature sources
FH3(TAMRA)	F:CTCCCACCCACAAATCCCTC	(ACC)7	231	[17]
	R:GGTCCTCCAAACTCTTCGCA			
FH7 (FAM)	F:AATCTTACTGGCGCGGGAAA R:GTGCTGCGGATTTCGATTCC	(GA)9	150	[17]
FH10 (FAM)	F:TGCTGGGGATAGGTCTTGGA	(TC)9TAGCTTCTT(TC)6	141	[17]
	R:AATATCCAGAGCCGAAGCCG			
FH21 (TAMRA)	F:AAGATCGTGGTGGTGAGCAG	(AGT)6	252	[17]
	R:CGTGGTGCTCACAAACCTTG			
FS4-11 (TAMRA)	F:AAGGCAACGGGGATAAAGTATTCA	(CGA)6	272 ~ 293	[15]
	R:CTCCGAGAGCAACTCCATCACG			
Frub38 (HEX)	F:ACACGTGCAGTGCTGCTGA	(AG)8AAC(GA)13	195–255	[14]
	R:ACAGCTGCCCAATTCCTTGA			
Frub398 (HEX)	F:GTACCTTAGATTCTAGTGTGAG	(GT)7ATA(TG)6C(GT)13	186–215	[14]
	R:TGGGATCTCATGAACTATTTAC			
Frub436 (FAM)	F:GTACTGTGATTAGTATCTTTGA	(AC)22	135–159	[14]
	R:CTAGCAATAACTCACTGATATTG			
MFC1 (HEX)	F:ACTAGACTGAAAAAACATTGC	(CT)13	192	[16]
	R: TGAGATTGAAAGGAAACGAG			

### Data analyses

For evaluating the polymorphism of the nine SSRs, the indexes of number of observed alleles (*N*a), number of effective alleles (*N*e), observed heterozygosity (*H*o) and expected heterozygosity (*H*e) for each locus were computed with GenAlEx software v.6.5 [42]. We also estimated the presence of stuttering, large allele dropout and null alleles using MICRO-CHECKER v.2.2.3 with a Bonferroni correction for multiple tests [43]. Hardy–Weinberg equilibrium (HWE) for each population over loci was assessed using online Genepop software v.4.7 (https://genepop.curtin.edu.au/) [44]. After that, a sequential Bonferroni correction for global tests was applied to the data from each population [45]. Wright’s *F*-statistics inbreeding coefficient (*F*_IS_) [46] for each population was estimated by Arlequin v.3.5 [47]. Usually, populations that have experienced a recent reduction of their effective population size may exhibit a correlative reduction of the allele numbers and heterozygosities at polymorphic loci [48]. The Bottleneck software v.1.2.02 [49] was used to detect genetic bottlenecks for each population. We used a two-phase model of mutation (TPM) [50] with 70% stepwise mutations and 30% multistep mutations, and a “Wilcoxon signed-rank test”, as recommended (for the polymorphic loci < 10) [49]. A significant level was set of 0.05.

To determine the population genetic structure, a Bayesian clustering approach was used with software of Structure v.2.3.4 [51]. The number of the most likelihood populations (*K*) was set from 1 to 8 and 5 iterations were run for each K. The 1,000,000 initial burn-in replications were followed by 1,000,000 Markov Chain Monte Carlo (MCMC) replications. The optimal K capturing the major structure of the populations of *F.* *hirta* was determined using Structure Harvester web v.0.6.94 (http://taylor0.biology.ucla.edu/structureHarvester/). The Nei’s genetic distances between populations were calculated in GenAlEx v.6.5, as an input for clustering analysis using Principal coordinate analysis (PCoA) implemented in GenAlEx v.6.5.

The analysis of molecular variance (AMOVA) was computed with significance determined by permutation test (1000 replicates) using Arlequin v.3.5 [47]. The pairwise standardized *G*-statistics genetic differentiation (*G’*_ST_) matrix [52] was computed by GenAlEx, then visualized by MEGA v.11 [53] program using the unweighted pair group method with an arithmetic mean (UPGMA) module, which would further determine the populations relationship of *F.* *hirta*.

Mantel test [54] was conducted by GenAlEx v.6.5 to determine whether there was a significant pattern of isolation by distcance (IBD) [55] between populations of *F.* *hirta*. Which estimate the correlations between two matrices, a matrix of standardized genetic differentiation (*G’*_ST_) computed by GenAlEx and a matrix of geographical distances (Km) between locations calculated using the Geographic Distance Matrix Generator v.1.2.3 [56]. Significance was determined with 999 permutations.

## Data Availability

The datasets generated and/or analyzed during the current study are available in the Dryad Digital Repository (https://datadryad.org/stash/share/82S4NX48s7uaI5vpGTjkYSoOsmd9ImdAd7ATz1kSPI4].

## Abbreviations

SSR: Simple Sequence Repeat
GAP: Good agricultural practice
*N*_a_: Number of observed alleles
*N*_e_: Number of effective alleles
*P*_Ar_: Private alleles
*I*: Shannon’s information index
*H*_o_: Observed heterozygosity
*H*_e_: Expected heterozygosity
*F*_IS_: Inbreeding coefficient
*PIC*: Polymorphism information content
TPM: Two-phase model of mutation
HWE: Hardy–Weinberg Equilibrium
MCMC: Markov chain Monte Carlo
UPGMA: Unweighted pair-group method of arithmetic averages
PCoA: Principal coordinate analysis
d.f.: Degree of freedom
*φ*_ST_: Genetic differentiation among populations within group
*G’*_ST_: Standardized genetic differentiation

## References

[1] Zhou ZK, Gilbert MG (2003). Flora of China (English Edition).

[2] Liu YR, Chen WN, Li F, Li C, Xie XN, Chao Z, Tian EW (2019). The complete chloroplast genome sequence of *Ficus*
*hirta* (Moraceae). Mitochondrial DNA B Resour.

[3] Food And Drug Administration Of Guangxi Zhuang Autonomous Region. Quality standard of Yao medicinal materials in Guangxi Zhuang Autonomous Region, vol. 1. Nanning: Guangxi Science&Technology Publishing House; 2014.

[4] Food and Drug Administration of Guangxi Zhuang Autonomous Region (2011). Quality standard of Zhuang medicine in Guangxi Zhuang Autonomous Region.

[5] Huang PW, Lu JQ, Lin H, Pang Y, Tang ML, Yu MP (2020). Advances in the study on the chemical composition, pharmacological effect and clinical application of Radix Fici Simplicissimae. J Liaoning Univ Tradit Chin Med.

[6] Liang Q, Dong W, Wang F, Wang W, Zhang J, Liu X (2021). *Ficus*
*hirta* Vahl. promotes antioxidant enzyme activity under ammonia stress by inhibiting miR-2765 expression in Penaeus vannamei. Ecotoxicol Environ Saf.

[7] Zhang WJ, Cao Y, Zhang Y, Ge Y, Wang S, Kang CZ, Wan XF, Xu HY, Guo LP (2021). Construction status and development strategy of GAP bases for Chinese herbal medicine. Zhongguo Zhong Yao Za Zhi.

[8] Zou RX (2019). Ultivation and Management Techniques of *Ficus*
*simplicissima* Lour in Imitating Wild Condition. Hortic Seed.

[9] Zhang X, Su HL, Yang J, Feng L, Li ZH, Zhao GF (2019). Population genetic structure, migration, and polyploidy origin of a medicinal species *Gynostemma*
*pentaphyllum* (Cucurbitaceae). Ecol Evol.

[10] Liu QQ, Lu ZY, He W, Li F, Chen WN, Li C, Chao Z, Tian EW (2020). Development and characterization of 16 novel microsatellite markers by Transcriptome sequencing for Angelica dahurica and test for cross-species amplification. BMC Plant Biol.

[11] Doebley JF, Gaut BS, Smith BD (2006). The molecular genetics of crop domestication. Cell.

[12] Mondal S, Rutkoski JE, Velu G, Singh PK, Crespo-Herrera LA, Guzmán C, Bhavani S, Lan C, He X, Singh RP (2016). Harnessing Diversity in Wheat to Enhance Grain Yield, Climate Resilience, Disease and Insect Pest Resistance and Nutrition Through Conventional and Modern Breeding Approaches. Front Plant Sci.

[13] Garrido-Cardenas JA, Mesa-Valle C, Manzano-Agugliaro F (2018). Trends in plant research using molecular markers. Planta.

[14] Crozier YC, Jia XC, Yao JY, Field AR, Cook JM, Crozier RH (2007). Microsatellite primers for *Ficus*
*racemosa* and *Ficus*
*rubiginosa*. Mol Ecol Notes.

[15] Zavodna M, Arens P, Van Dijk PJ, Partomihardjo T, Vosman B, Van Damme JMM (2005). Pollinating fig wasps: genetic consequences of island recolonization. J Evolution Biol.

[16] Khadari B, Hochu I, Santoni S, Kjellberg F (2001). Identification and characterization of microsatellite loci in the common fig (*Ficus*
*carica* L.) and representative species of the genus Ficus. Mol Ecol Notes.

[17] Zheng LN, Nason JD, Liang D, Ge XJ, Yu H (2015). Development and characterization of microsatellite loci for *Ficus*
*hirta* (Moraceae). Appl Plant Sci..

[18] Chinese Herbalism Editorial Board SOTP (1999). Chinese Herbalism.

[19] Liu XF, Ma YP, Wan YM, Li ZH, Ma H (2020). Genetic diversity of *phyllanthus*
*emblica* from two different climate type areas. Front Plant Sci.

[20] Govindaraj M, Vetriventhan M, Srinivasan M (2015). Importance of genetic diversity assessment in crop plants and its recent advances: an overview of its analytical perspectives. Genet Res Int.

[21] Zhong YC, Wang Y, Sun ZM, Niu J, Shi YL, Huang KY, Chen J, Chen JH, Luan MB (2021). Genetic Diversity of a Natural Population of Akebia trifoliata (Thunb.) Koidz and Extraction of a Core Collection Using Simple Sequence Repeat Markers. Front Genet.

[22] Zhang J, Jiang K, Shi YS, Liu M, Chen XY (2011). Development and polymorphism of microsatellite primers in *Ficus*
*pumila* L. (Moraceae). Am J Bot..

[23] Essid A, Aljane F, Neily MH, Ferchichi A, Hormaza JI (2021). Assessment of genetic diversity of thirty Tunisian fig (*Ficus*
*carica* L.) accessions using pomological traits and SSR markers. Mol Biol Rep.

[24] Yu H, Nason JD (2013). Nuclear and chloroplast DNA phylogeography of *Ficus*
*hirta*: obligate pollination mutualism and constraints on range expansion in response to climate change. New Phytol.

[25] Yu H, Nason JD, Ge X, Zeng J (2010). Slatkin's Paradox: when direct observation and realized gene flow disagree. A case study in Ficus. Mol Ecol.

[26] Brandström M, Ellegren H (2008). Genome-wide analysis of microsatellite polymorphism in chicken circumventing the ascertainment bias. Genome Res.

[27] Yuan N, Li M, Jia C (2020). De novo transcriptome assembly and population genetic analyses of an important coastal shrub, Apocynum venetum L. BMC Plant Biol.

[28] Wu FF, Ma SN, Zhou J, Han CY, Hu RC, Yang XY, Nie G, Zhang XQ (2021). Genetic diversity and population structure analysis in a large collection of white clover (*Trifolium*
*repens* L.) germplasm worldwide. Peer J.

[29] Hamrick JL, Godt MJW (1996). Effects of life history traits on genetic diversity in plant species. Philos Trans R Soc B.

[30] Heer K, Kalko EK, Albrecht L, García-Villacorta R, Staeps FC, Herre EA, Dick CW (2015). Spatial scales of genetic structure in free-standing and strangler Figs (*Ficus*, Moraceae) inhabiting neotropical forests. PLoS ONE.

[31] Tian S, Kou Y, Zhang Z, Yuan L, Li D, López-Pujol J, Fan D, Zhang Z (2018). Phylogeography of *Eomecon*
*chionantha* in subtropical China: the dual roles of the Nanling Mountains as a glacial refugium and a dispersal corridor. BMC Evol Biol.

[32] Liu WZ, Xie JG, Zhou H, Kong HH, Hao G, Fritsch PW, Gong W (2021). Population dynamics linked to glacial cycles in *Cercis*
*chuniana* F. P. Metcalf (Fabaceae) endemic to the montane regions of subtropical China. Evol Appl.

[33] Holsinger KE, Weir BS (2009). Genetics in geographically structured populations: defining, estimating and interpreting *F*(ST). Nat Rev Genet.

[34] Lomáscolo SB, Levey DJ, Kimball RT, Bolker BM, Alborn HT (2010). Dispersers shape fruit diversity in *Ficus* (Moraceae). P Natl Acad Sci USA.

[35] Huang JF, Darwell CT, Peng YQ (2021). Homogenized phylogeographic structure across the indo-burma ranges of a large monoecious fig, *ficus*
*altissima* blume. Diversity.

[36] Tian EW, Nason JD, Machado CA, Zheng LN, Yu H, Kjellberg F (2015). Lack of genetic isolation by distance, similar genetic structuring but different demographic histories in a fig-pollinating wasp mutualism. Mol Ecol.

[37] Ahmed S, Compton SG, Butlin RK, Gilmartin PM (2009). Wind-borne insects mediate directional pollen transfer between desert fig trees 160 kilometers apart. Proc Natl Acad Sci U S A.

[38] Liu M, Zhang J, Chen Y, Compton SG, Chen XY (2013). Contrasting genetic responses to population fragmentation in a coevolving fig and fig wasp across a mainland–island archipelago. Mol Ecol.

[39] Dong YB, Pei XW, Yuan QH, Wang F, Wu HJ, Jia SR, Peng YF (2012). Genetic differentiation of *Oryza*
*ruffipogon* Griff. from Hainan Island and Guangdong, China based on Hd1 and Ehd1 genes. Biochem Syst Ecol.

[40] Chung MY, Son S, Herrando-Moraira S, Tang CQ, Maki M, Kim YD, López-Pujol J, Hamrick JL, Chung MG (2020). Incorporating differences between genetic diversity of trees and herbaceous plants in conservation strategies. Conserv Biol.

[41] Yang JB, Li DZ, Li HT (2014). Highly effective sequencing whole chloroplast genomes of angiosperms by nine novel universal primer pairs. Mol Ecol Resour.

[42] Peakall R, Smouse PE (2012). GenAlEx 6.5: genetic analysis in Excel. Population genetic software for teaching and research–an update. Bioinformatics.

[43] Van Oosterhout C, Hutchinson WF, Wills DPM, Shipley P (2004). micro-checker: software for identifying and correcting genotyping errors in microsatellite data. Mol Ecol Notes.

[44] Mahesh BA, Kannan E, Davis G, Venkatesan P, Ragunath PK (2020). GenPop-An Online Tool to Analyze Human Population Genetic Data. Bioinformation.

[45] Rice WR (1989). Analyzing tables of statistical tests. Evolution.

[46] Weir BS, Cockerham CC (1984). Estimating F-statistics for the analysis of population structure. Evolution.

[47] Excoffier L, Laval G, Schneider S (2007). Arlequin (version 3.0): an integrated software package for population genetics data analysis. Evol Bioinform Online.

[48] Jarvis JP, Cropp SN, Vaughn TT, Pletscher LS, King-Ellison K, Adams-Hunt E, Erickson C, Cheverud JM (2011). The effect of a population bottleneck on the evolution of genetic variance/covariance structure. J Evol Biol.

[49] Piry S, Luikart G, Cornuet J (1999). Computer note. BOTTLENECK: a computer program for detecting recent reductions in the effective size using allele frequency data. J Hered.

[50] Di Rienzo A, Peterson AC, Garza JC, Valdes AM, Slatkin M, Freimer NB (1994). Mutational processes of simple-sequence repeat loci in human populations. Proc Natl Acad Sci U S A.

[51] Pritchard JK, Stephens M, Donnelly P (2000). Inference of population structure using multilocus genotype data. Genetics.

[52] Hedrick PW (2005). A standardized genetic differentiation measure. Evolution.

[53] Kumar S, Stecher G, Li M, Knyaz C, Tamura K (2018). MEGA X: Molecular Evolutionary Genetics Analysis across Computing Platforms. Mol Biol Evol.

[54] Wright S (1943). Isolation by Distance. Genetics.

[55] Rousset F (1997). Genetic differentiation and estimation of gene flow from F-statistics under isolation by distance. Genetics.

[56] Ersts PJ. Geographic Distance Matrix Generator (version 1.2.3). American Museum of Natural History, Center for Biodiversity and Conservation. Available from http://biodiversityinformatics.amnh.org/open_source/gdmg. Accessed on 2022-7-9.

